# Effects of Adriamycin-Cytoxan chemotherapy on hematological and electrolyte parameters among breast cancer patients

**DOI:** 10.3389/fonc.2023.1103013

**Published:** 2023-05-02

**Authors:** Fikremariam Abiye Tadesse, Abebaye Aragaw Leminie

**Affiliations:** Department of Physiology, School of Medicine, College of Health Sciences, Addis Ababa University, Addis Ababa, Ethiopia

**Keywords:** breast cancer, Adriamycin-Cytoxan, effects, hematological indices, serum electrolyte

## Abstract

**Background:**

Adriamycin-Cytoxan (AC) is a common chemotherapy treatment for breast cancer (BC) patients. Its electrolyte and hematological adverse effects have not been addressed adequately.

**Objective:**

This study aimed to assess the effect of AC on hematological and electrolyte parameters among BC patients.

**Methods:**

A hospital-based comparative cross-sectional study design was conducted from March to November 2022. Randomly selected AC-treated (n=100) and untreated (n=100) patients were included. Structured questionnaire and medical records were used to collect sociodemographic data. Anthropometric parameters, hematological indices, and serum electrolytes were measured. Cobas Integra 400^+^and SYSMEX-XT-4000i were used to analyze serum electrolytes and hematological indices respectively. The data were analyzed using SPSS version 25. Independent t-test and chi-square test were used. *p-value <*0.05 was considered statistically significant.

**Results:**

AC-treated patients’ mean total white blood cell (TWBC), neutrophil (NE), lymphocyte (LY), red blood cell (RBC), hemoglobin (Hgb), hematocrit (HCT), and sodium(Na^+^) values were significantly reduced (p<0.05) than patients with no treatment. However, mean eosinophils (EO), platelet (PLT) counts, red cell distribution-width (RDW), potassium (K^+^), and plateletcrit (PCT values were significantly increased (p<0.05).

**Conclusion:**

The majority of blood cells and serum sodium were affected by AC treatment. Incorporating these parameters in the routine analysis and further studies on the detailed mechanism of action of this drug is required.

## 1 Background

BC in women is a highly injurious tumor originating from the breast tissue and is becoming prevalent among Ethiopian women and is treated by chemotherapy with a high risk of recurrence (1). However, this therapy results in serious and life-threatening adverse effects. Adriamycin-Cytoxan (AC) is combination drugs are commonly used. Bone marrow, gastrointestinal, renal, musculoskeletal, and nervous systems are negatively affected (2). In this case, dose reduction, dose delays, treatment cessation, mortality, and morbidity are frequent.

Cells sharing tumor cells features and divided highly such as bone marrow, digestive tract, urinary tract, hair follicles, and reproductive cells are strongly affected by AC therapy (3–6). This indicates that AC treatment could alter hematological indices and serum electrolytes. However, studies done on the hematological and electrolyte adverse effects of this therapy in Ethiopia are insufficient. This study is, therefore, aimed to determine the effect of AC chemotherapy on hematological and serum electrolytes in patients with BC.

## 2 Materials and methods

### 2.1 Study area

The study was conducted at Addis Ababa University, College of Health Sciences, Black Lion Specialized Teaching Hospital (BLSTH), and Saint Paul’s Hospital Millennium Medical College (SPHMMC), Addis Ababa, Ethiopia. BLSTH is one of the oldest and largest teaching hospitals and SPHMMC is the second-largest hospital in Addis Ababa, Ethiopia. The oncology units of BLSTH and SPHMMC give inpatient and outpatient services for BC patients from all over regions in Ethiopia.

### 2.2 Study design and study period

Comparative cross-sectional study design was conducted from March to November 2022.

### 2.3 Source and study population

All female BC patients who visited BLSTH and SPHMMC during the study period were the source population in this study. All female BC patients with histopathological evidence and who fulfilled inclusion criteria were the study population. Those patients who voluntarily consented to participate in the study and attend regular follow-ups in the oncology clinic of BLSTH and SPHMMC during the study period participated in this study.

### 2.4 Sample size determination and sampling technique

Convenient sample size determination approach was used to determine the number of BC patients in this study. A total of 200 BC patients were recruited for the study. Samples were collected from randomly selected patients until the required sample size was obtained. The two hospitals were selected purposively based on the high patient flow rate.

### 2.5 Inclusion and exclusion criteria

All volunteer and newly diagnosed BC patients who have not yet taken AC and those who have taken and completed the 3^rd^ cycle of AC were involved in this study. Study patients with any known metabolic, GIT, renal, cardiovascular, and hematological disorders were excluded from this study. In addition, patients with chronic alcohol intake, smoking cigarettes, taking any form of drugs for any known acute and chronic illness, vomiting, diarrhea, contraceptive pills, pregnancy, and other co-morbidities were not included. Patients who were unable to give written consent were also excluded.

### 2.6 Data collecting tools

Structured and pre-tested questionnaire, physical measurements, and blood analysis were used to collect data. The questionnaire used to collect socio-demographic and maternal-related information was written in English and then translated into Amharic. The response from each participant was translated back into English to assure its accuracy and analysis. Blood pressure and BMI (weight/m^2^) were collected using physical measurements. Blood analysis was used to determine electrolytes and hematological indices.

### 2.7 Complete blood cell count and biochemical analysis

8 ml of brachial venous blood was collected by three trained nurses after informed consent was obtained from each participant. 4ml of blood was placed into an EDTA tube to determine WBCs, RBC, PLT, Hgb, HCT, RDW, MCV, MCH, MPV, PCT, and MCHC using CBC machine (SYSMEX-XT-4000i). The remaining 4 ml of blood was placed into a serum tube and centrifuged at 6000 rpm for 10 minutes. The serum was separated and lowly transferred into new Eppendorf tubes to be preserved in a refrigerator at -20 °C until used. Na^+^, K^+^, and Ca^+2^ were analyzed using Cobas Integra 400^+^) (7).

### 2.8 Ethical consideration

An ethical approval letter was obtained from the Department of Medical Physiology (reference: MP/09/22), Faculty of Medicine, College of Health Sciences, Addis Ababa University. Official letters were written to SPHMMC and BLSTH oncology from the Department of Medical Physiology. During data collection, written consent was obtained from each participant.

### 2.9 Data analysis

The data were manually checked for clarity and completeness, coded, cleaned, and entered into the SPSS version 25.0. An independent t-test and chi-square test statistics were used to compare the mean difference for continuous and categorical variables respectively between the groups. Percentage and mean±SD were used in the descriptive analysis and p-value < 0.05 at 95% CI was considered statistically significant.

### 3 Results

#### 3.1 Socio-demographic characteristics of patients

In this study, significant mean age difference between AC-treated and untreated BC patients was not observed (47.95 ± 13.77 vs 44.54 ± 11.84; p =0.062). The larger proportion of the study participants was the aged between 45-59 (n=73), while the least was under 30 years old (n=16) (**Table 1**).

**Table 1:** Socio-demographic characteristics of AC-treated and untreated BC patients.

Characteristics	Categories	Untreated (n=100)	Treated (n=100)	P-value
Age (in years), n (%)	≤30	7 (7)	9 (9)	0.603
	31-44	39 (39)	32 (32)	
	45-59	37 (37)	36 (36)	
	≥60	17 (17)	23 (23)	
Residence, n (%)	Rural	37 (37)	33 (33)	0.553
	Urban	63 (63)	67 (67)	
Marital status, n (%)	Single	29 (29)	24 (24)	0.518
	Married	51 (51)	51 (51)	
	Divorced	6 (6)	4 (4)	
	Windowed	14 (14)	21 (21)	
Educational status, n (%)	No formal education	11 (11)	15 (15)	0.813
	Elementary	19 (19)	18 (18)	
	High school	16 (16)	13 (13)	
	College and above	54 (54)	54 (54)	
Religion, n (%)	Muslim	31 (31)	26 (26)	0.434
	Christian	69 (69)	74 (74)	
Menopausal status, n (%)	Premenopausal	58 (58)	54 (54)	0.569
	Postmenopausal	42 (42)	46 (46)	

AC, Adriamycin-Cytoxan; BC, breast cancer; p-value of ≤0.05 implies a significant difference between categories.

The majority of the treated (67%) and untreated (63%) patients were from urban areas. More than half of the treated (51) and untreated (51%) patients were married. Moreover, 74% of the treated and 69% of untreated were Christian, while 54% of the treated and 58% of untreated patients were in the premenopausal stage (**Table 1**).

#### 3.2 Clinical characteristics of patients

Significant difference in mean BMI between treated and untreated participants was not observed (25.13±4.0 Kg/m^2^ vs 24.22±3.7 kg/m2, p = 0.100). The larger proportion of treated (70%) and untreated (65%) patients had BMI in the normal range. 56% of the treated and 57% of untreated patients were at stages I and II. The remaining 44% of the treated and 43% of untreated patients were at stages III and IV. There was no significant difference in mean SBP (119.42±1.87 vs 118.9±1.9; *p =* 0.060 and DBP (79.0 ± 2.13 vs 78.6±2.1; *p* = 0.182) between untreated and treated patients (**Table 2**).

**Table 2:** Clinical characteristics of AC-treated and untreated BC patients.

Characteristics	Categories	Untreated (n=100)	Treated (n=100)	P-value
WHO clinical stage, n (%)	Stage 1	26 (26)	22 (22)	0.705
	Stage 2	31 (31)	34 (34)	
	Stage 3	21 (21)	26 (26)	
	Stage 4	22 (22)	18 (18)	
Metastasis, n (%)	Yes	22 (22)	17 (18)	0.372
	No	78 (78)	83 (82)	
BMI, n (%)	<18 Kg/m^2^	5 (5)	6 (6)	0.793
	18-24.5 Kg/m^2^	65 (65)	70 (70)	
	25-29.5 Kg/m^2^	15 (15)	11 (11)	
	≥30 Kg/m^2^	15 (15)	13 (13)	
BMI (Mean ± SD)		25.13 ± 4.0	24.22 ± 3.7	0.100
BP (Mean ± SD)	SBP (mmHg)	119.42 ± 1.87	118.9 ± 1.9	0.060
	DBP (mmHg)	79.0 ± 2.13	78.6 ± 2.1	0.182

AC, Adriamycin-Cytoxan; BC, breast cancer; BMI, Body Mass Index; SBP, Systolic Blood Pressure; DBP, Diastolic Blood Pressure; and SD, Standard Deviation. Categorical variables were also expressed as numbers (n) and percentages (%). Continuous variables were expressed as mean ± standard deviation (SD). P-value of < 0.05 implies a significant difference between groups.

#### 3.3 Comparison of hematological and electrolyte parameters

Mean TWBC (8.47±1.85.12/*µ*L vs 5.26±1.52/*µ*L; *p* < 0.0001), NE (4.13±1.48/*µ*L vs 2.12±.81/*µ*L); *p* < 0.0001) and LY (2.74±1.33/*µ*L vs 1.60±.88/*µ*L; *p* < 0.0001) counts of the untreated patients were significantly higher than treated patients. However, the mean EO count was significantly less in untreated patients than in the treated patients (.58±.40/*µ*L vs.75±.46/*µ*L); *p* = 0.006).

Mean RBC (4.52±.53/*µ*L vs 4.06±.64/*µ*L; *p* < 0.0001), Hgb (12.49±1.29g/dL vs 11.08±.84g/dL; *p* < 0.0001) and HCT (36.99±3.98% vs 33.01±2.31%; p < 0.0001) values of the untreated patients were significantly higher than the treated patients. Mean RDW (12.86±1.79 vs 18.39±2.77%; *p* < 0.0001) was significantly less in untreated patients compared to the treated patients. The mean PLT (245.68±76.16/*µ*L vs 373.88±147.99/*µ*L; *p* < 0.0001), and PCT (0.13±.04% vs 0.22±.08%; *p* < 0.0001) values of the untreated patients were significantly lower than the treated patients.

No significant differences in mean MPV (8.23±1.14% vs 7.96±1.15%; p = 0.092), MO (.86±.37/*µ*L vs.82±.34/*µ*L; *p* = 0.498), and BA (0.13/*µ*L±.12/*µ*L vs 0.14±.15/*µ*L; p = 0.721) values were observed between treated and untreated groups. Significant differences in the mean MCV (82.56±8.15 fL vs 82.70±10.09 fL; *p* = 0.912), MCH (27.91±2.65pg vs 27.70±3.33pg; p = 0.630), and MCHC (33.81±.94/dL vs 33.54±1.15g/dL; *p* = 0.075) values were not also observed between treated and untreated groups.

In this study, the mean serum Na^+^ (142.43±4.47 mmol/L vs 133.34±6.11 mmol/L; *p* < 0.0001) level in the untreated patients was significantly higher than the treated group, while serum K^+^ (3.81±.50 and vs 5.16±.98 mmol/L; *p* < 0.0001 level was significantly increased in the treated group.

However, no significant difference in the serum Ca^2+^ (8.95±.97mg/dL vs 8.78±1.00mg/dL; *p* = 0.230) level was observed between treated and untreated patients (**Table 3**).

**Table 3:** Comparison of hematological and electrolyte parameters between AC-treated and untreated BC patients.

Variables (Mean ± SD)	Untreated (n=100)	Treated (n=100)	p-value
TWBC (/*µ*L)	8.47±1.85	5.26±1.52	<0.0001
NE (/*µ*L)	4.13±1.48	2.12±.81	<0.0001
LY (/*µ*L)	2.74±1.33	1.60±.88	<0.0001
MO (/*µ*L)	0.86±.37	0.82±.34	0.498
EO (/*µ*L)	0.58±.40	0.75±.46	0.006
BA (/*µ*L)	0.13±.12	0.14±.15	0.721
RBC (/*µ*L)	4.52±.53	4.06±.64	<0.0001
Hgb (g/dL)	12.49±1.29	11.08±.84	<0.0001
HCT (%)	36.99±3.98	33.01±2.31	<0.0001
RDW (%)	12.86±1.79	18.39±2.77	<0.0001
MCV (fL)	81.96±7.86	80.13±8.35	0.113
MCH (pg)	27.91±2.65	27.70±3.33	0.630
MCHC (g/dL)	33.61±1.12	33.54±1.15	0.648
PLT(/*µ*L)	245.68±76.16	373.88±147.99	<0.0001
MPV (%)	8.23±1.14	7.96±1.15	0.092
PCT (%)	0.13±.04	0.22±.08	<0.0001
Na^+^(mmol/L)	142.43±4.47	133.34±6.11	<0.0001
Ca^2+^(mg/dL)	8.62±.89	8.75±1.01	0.330
K^+^ (mmol/L)	3.81±.50	5.22±.107	<0.0001

TWBC, Total White Blood Cell; NE, Neutrophil; LY, Lymphocyte; MO, Monocyte; EO, Eosinophils; BA, Basophil; RBC, Red Blood Cell; Hgb, Hemoglobin; HCT, Hematocrit; RDW, Red Cell Distribution Width; MCV, Mean Cell Volume; MCH, Mean Cell Hemoglobin; MCHC, Mean Cell Hemoglobin Concentration; PLT, Platelet; MPV, Mean Platelet Volume; PCT, Plateletcrit, Na^+^: Sodium, Ca^2+^: Calcium, K^+^: Potassium, BC: breast cancer, AC: Adriamycin-Cytoxan. Continuous variables were expressed as mean ± standard deviation (SD) and p-value < 0.05 implies a significant difference between the two study group’s variables.

#### 3.4 Comparison of hematological and electrolyte parameters based on clinical stages

The mean TWBC (5.64±1.43 vs 8.87±1.73; *p* < 0.0001 and 4.77 ± 1.52 vs 7.94 ± 1.89; *p* < 0.0001), NE (2.27±.82 vs 4.41 ± 1.48; *p* < 0.0001 and 1.93 ±.76 vs 3.75 ± 1.42; *p* > 0.001) and LY (1.83±.94 vs 2.74 ± 1.42; *p* < 0.0001 and 1.31 ±.71 vs 2.74 ± 1.22; *p* < 0.0001) of treated patients was significantly reduced as compared with untreated patients in lower and advanced stages of the disease, respectively. Other hematological indices and serum electrolytes were also significantly affected in both the lower and the advanced stages of cancer (**Table 4**).

**Table 4:** Comparison of hematological and electrolyte parameters based on clinical stages.

Parameter (Mean ± SD)	Stage I and II (lower stage)			Stage III and IV (advanced stage)		
	Untreated (n=57)	Treated (n=56)	p-value	Untreated (n=43)	Treated (n=44)	p-value
TWBC (/*µ*L)	8.87±1.73	5.64±1.43	0.000	7.94±1.89	4.77±1.52	<0.0001
NE (/*µ*L)	4.41±1.48	2.27±.82	0.000	3.75±1.42	1.93±.76	<0.0001
LY(/*µ*L)	2.74±1.42	1.83±.94	0.000	2.74±1.22	1.31±.71	<0.0001
MO(/*µ*L)	0.83±.37	0.89±.36	0.415	0.81±.38	0.82±.30	0.929
EO(/*µ*L)	0.63±.44	0.76±.48	0.150	0.50±.33	0.73±.43	0.007
BA(/*µ*L)	0.14±.10	0.13±.17	0.965	0.13±.15	0.15±.13	0.559
RBC(/*µ*L)	4.54±.54	4.29±.58	0.020	4.49±.51	3.77±.60	<0.0001
Hgb (g/dL)	12.66±1.43	11.49±.71	0.000	12.27±1.05	10.56±.71	<0.0001
HCT (%)	37.76±4.34	34.16±1.92	0.000	35.98±3.23	31.56±1.93	<0.0001
RDW (%)	12.96±2.06	17.75±2.44	0.000	12.71±1.36	19.21±2.97	<0.0001
MCV (fL)	82.94±7.57	80.70±9.35	0.164	80.66±8.13	79.41±6.91	0.440
MCH (pg)	28.22±2.59	27.13±3.26	0.051	27.49±2.7	28.44±3.30	0.148
MCHC(g/dL)	33.67±.93	33.63±1.06	0.830	33.54±1.35	33.43±1.25	0.687
PLT(/*µ*L)	272.0±81.9	313.1±119.7	0.036	210.77±50.4	451.2±145.5	<0.0001
MPV (%)	8.28±1.11	8.20±.99	0.676	8.16±1.18	7.65±1.26	0.054
PCT (%)	0.14±.04	0.19±.08	0.000	0.13±.03	0.26±.08	<0.0001
Na^+^(mmol/L)	142.9±4.58	133.92±4.48	0.000	141.76±4.29	132.59±7.71	<0.0001
K^+^ (mmol/L)	3.76±.52	4.95±.94	0.000	3.87±.48	5.57±1.13	<0.0001
Ca^+2^ (mg/dL)	8.82±1.03	8.93±.78	0.545	8.35±.55	8.52±1.22	0.385

TWBC, Total White Blood Cell; NE, Neutrophil; LY, Lymphocyte; MO, Monocyte; EO, Eosinophils; BA, Basophil; RBC, Red Blood Cell; Hgb, Hemoglobin; HCT, Hematocrit; RDW, Red Cell Distribution Width; MCV, Mean Cell Volume; MCH, Mean Cell Hemoglobin; MCHC, Mean Cell Hemoglobin Concentration; PLT, Platelet; MPV, Mean Platelet Volume; PCT, Plateletcrit; Na^+^, Sodium; Ca^2+^, Calcium; K^+^, Potassium; AC, Adriamycin-Cytoxan. Continuous variables were expressed as mean ± standard deviation (SD) and p-value < 0.05 implies a significant difference between the two study group’s variables.

## 4 Discussion

In the present study,200 patients, 100 of 3^rd^ cycle AC treated and 100 AC untreated BC patients were included to compare hematological and serum electrolyte parameters between the groups.AC treatment hematological toxicity and electrolyte disorders are very common complications among women with BC (8). They associated with severe clinical manifestations that worsen patient’s clinical condition or outcome up to more serious life-threatening events that influencing quality of life (8).

The socio-demographic data revealed that most study patients were found to age between 31 and 59 years. This report supported by findings from other local studies conducted in Addis Ababa, TASH, and 60% (9) and 67% (10). In contrast, global research from Western countries normally demonstrated that the highest prevalence of BC is reported at the age of above 65 (11, 12). The findings indicated that BC developing at a lower age in Ethiopia is higher than in Western countries. This could be attributed to BC awareness and ignoring the warning signs, and other predisposing factors such as reproductive history, on-cancerous breast diseases, and family history of BC, previous radiotherapy treatments, and exposure to other drugs. Racial differences in the production of endogenous hormones, existent with higher younger population age groups in Ethiopia also could contribute to this age difference at which BC is developed. Africans had higher levels of estradiol, free estradiol, higher IGF-1, and lower level of sex hormone binding globulin (SHBG), as it is well known that SHBG is associated with a decrease in the risk of BC (13, 14). Although, urban population has advanced education and better awareness about BC, financial capacity, and easier access to early stage diagnosis, the majority of the patients were reported to be from urban areas in the country. The reason might be attributed to the lifestyle, behavioral changes and reproductive factors. Previous studies indicated that lifestyle and reproductive factors such as low fertility rate in urban populations have very remarkable effects on BC development (9, 15, 16). However, AC treated and untreated study groups were comparable in all of the socio-demographic and clinical characteristics, indicating that variations in the hematological and serum electrolytes parameters could attributed to the AC-treatment.

A combination of Adriamycin with Cytoxan (AC) is a commonly used chemotherapy to treat BC (17, 18). Cyclophosphamide is a prodrug and needs to be metabolized by the cytochrome P450 system to active metabolites that prevent cell division by cross-linking DNA strands and decreasing DNA synthesis of cancerous cells (17, 18). However, some of the normal cells or non-targeted cells are also sensitive to this treatment and get damaged (19).

In our study, there was a significant decrement in mean TWB count among BC patients with the 3^rd^ cycle AC treatment. This finding is in line with studies conducted before (20–23). According to a previous study, AC treatment promotes oxidative stress in the bone marrow (24). Under oxidative stress, reactive oxygen species (ROS) are generated at high levels and induce cellular damage and death (25–27). Oxidative stress not only produces direct and acute cell damage but also affects cellular metabolism and disturbs the balance of redox reactions (28). In due course, leading to a persistent and prolonged increase in ROS production, which is capable of causing oxidative damage to hematopoietic stem cells (28). This indicates that although further studies in the mechanisms of AC treatment induction of hematopoiesis alteration are required, oxidative stress induced by AC treatment could attribute to the reduction of TWB count in our study.

Similar to the previous studies (21, 22), there was a significant reduction in neutrophil count among BC patients who completed the 3^rd^ cycle of AC chemotherapy treatment. According to previous studies, neutropenia is the most common toxicity seen in patients undergoing systemic AC chemotherapy treatment (26–28). The reduction of neutrophils in this study could attribute to impairment in hematopoiesis. Anticancer drugs bind covalently with bone marrow DNA of cells to form intra- and inter-strand cross-links of DNA that cause DNA damage during its replication and impair protein function (29, 30). Progressive depletion of hematopoietic stem cells in the bone marrow prevents cell growth which causes cell death and reduced white cell indices (29, 30). The gradual exhaustion of the stem cell in the bone marrow eventually inhibits the body to produce healthy WBCs (31).

In this study, the lymphocyte count was significantly reduced in the treatment group. Studies conducted before also revealed the reduction of lymphocytes (30–32). As reported in the previous studies (32, 33), this finding in our study indicates, chemotherapy is also associated with adverse effects on non-target tissues and affects immune responses.

The therapy compromises the innate and adaptive immune responses by influencing the homeostasis of the hematopoietic compartment through lymph depletion (32, 33). Hematopoietic DNA replication of cells is impaired (29, 30). Progressive depletion of hematopoietic stem cells in the bone marrow prevents cell growth which causes immune cell death and reduction of white blood cells (29, 30).

The eosinophil count was increased in the treatment group of BC patients and could be attributed to the inflammatory effects of the therapy. Poncin et al. reported that rapidly developing drug-related eosinophilia is often seen in the presence of an allergic reaction, aggressive inflammation, or malignancy (25). Poncin et al. (25) also revealed that eosinophils are involved in the immune response to breast tumors. Various studies also reported that platelets release chemokines in response to drug toxicity and increase eosinophil counts through chemicals (eosinophils activating factor, Platelet P-selectin) released from platelets (34, 35). These chemicals inhibit Eosinophils apoptosis and prolong survival (34, 35).

Contrarily, significant differences in monocyte and basophil were not observed between treated and untreated BC patients. This finding is in line with studies conducted previously (22, 23). However, another study (21) showed that a significant difference in monocyte and basophil count was observed after 3^rd^ cycle of AC treatment. The possible reason for this discrepancy could be attributed to the difference in the machine used. Kurup et al. (21) analyzed CBC using Beck man and Coulter CBC machine, while SYSMEX-XT-4000i was used in our study. The difference in the number of stages of cancer and sample size could also be attributed to the difference in the findings in that in our study, 20% were an advanced stage, and the number of BC patients who completed the 3^rd^ cycle AC- treatment was 100, while 27.2% of the patients were in the advanced stage with the sample size of184 in the study conducted by Kurup et al. (21).

The present study also demonstrated that RBCs, hemoglobin, and HCT values were significantly reduced in AC-treated patients compared to untreated ones. These findings are in agreement with studies done previously (22, 23, 36, 37). The decrease in these red cell indices might be the consequence of treatment-induced oxidative stress erythropoiesis failure and destruction of mature cells. Blockage of the incorporation of iron into hemoglobin due to a disturbance in the bio-generation structure of hemoglobin molecules and oxidation of irons causes hemoglobin synthesis problems (38).

Chemotherapy promotes inflammatory cytokine production, which suppresses erythropoietin (EPO) production and erythropoiesis (39). Mean RBC count showed a high decline after chemotherapy processes (39). This could be due to increased levels of pro-inflammatory cytokines, such as IL-1, IL-6, and TNF-α, and INF-δ that induce iron retention by the reticuloendothelial system, gastrointestinal tract, and liver, thereby exerting an inhibitory effect on erythroid precursors. An earlier report revealed that the low level of these parameters is also associated with bone marrow suppression attributed to cancer (40).

Similar to the previous study (23), the RDW value of AC treated group was significantly higher than the untreated group in our study. High RDW values are associated with drug-induced inflammation, oxidative stress, and disrupted erythropoiesis (24). RDW is an indicator of impaired erythropoiesis and abnormal red blood cell survival (24). According to Taherkhani et al. (13), the size variability in RBCs, RDW, is correlated with oxidative stress-induced inflammation, nutrition, and impaired renal function, with inadequate production of EPO.

In line with previous study findings (23, 37), significant differences in mean MPV, MCV, MCH, and MCHC values were not observed before AC treatment and after the completion of the 3^rd^ cycle of AC treatment. This indicates that the AC treatment didn’t affect all blood cell indices.

Regarding the platelet indices, platelet count and plateletcrit were significantly increased among AC treated group. Other studies also showed the increment of these parameters among AC-treated BC patients (21–23, 41). An earlier study (40), suggested that the increase in these platelet indices could be attributed to reactive thrombocytosis caused by AC treatment-induced anemia. On the other hand, increased platelet and PCT are indicators of enhanced platelet activities (42), organ inflammation (43), anxiety, and depression-like symptoms (44), indicating that AC treatment aggravates organ inflammation and anxiety disorders. Besides, systemic inflammation leads to the release of several pro-inflammatory mediators, interleukin IL-1, IL-3 and IL-6, which stimulate megakaryocyte proliferation (45). When cells are damaged by anticancer drugs, they release chemicals that trigger a response from our immune system (46). These inflammatory cytokines stimulate the process of platelet production by megakaryocytes in the bone marrow (46). This, further, platelet activation leads to the release of more cytotoxic mediators, such as reactive oxygen metabolites, free radicals, and cationic proteins (46).

Unlike our study, a study conducted by Steph et al. (20) revealed that significant differences in the platelet and plateletcrit were not observed between treated and untreated BC patients. The difference in study design, stage of cancer, supplementations, drug dose, and cut-off values between the two study groups could be the possible reasons for the inconsistent findings.

Although a significant difference in the serum calcium level was not found between treated and untreated patients in the current study, a study conducted by Hassan et al. (37) revealed that there was a significant reduction in the mean serum calcium level after the completion of 3^rd^ cycle of AC treatment. The possible reason for this disagreement could be due to differences in dietary habits, stage of cancer, age, cut-off values, and genetic makeup. However, the serum sodium level was significantly reduced among AC-treated BC patients in this study. This finding is consistent with a study conducted before (21). The reduction in the serum sodium level may be due to the effect of chemotherapeutic drugs that impair the kidney’s ability to handle sodium balance. A previous study revealed that the oxidative stress induced by chemotherapy was the reason for the reduction of serum sodium level (47).

Adriamycin possesses an anthracycline skeleton and generates ROS that leads to DNA damage of renal epithelial cells involved in electrolyte handling (15, 48). These anticancer drugs often cause a variety of adverse events and induction of ROS (49). Oxidative stress inhibits Na^+^-K^+^ ATPase and sodium ion channels in the apical membrane of renal tubular epithelial cells (50). Kidneys could be vulnerable to the development of drug toxicity due to their role in the metabolism and excretion of toxic agents. The proximal segment of a nephron, in particular, has a significant capacity for the uptake of drugs *via* endocytosis or transporter proteins (15, 51). The high rate of delivery and uptake results in high intracellular concentrations of drug metabolites and renal expression of tumor necrosis factor ***a*** that leads to the formation of potentially toxic metabolites and ROS (15). Oxidative stress also causes mitochondrial dysfunction, decreased ATPase activity, impaired solute transport, and altered cation balance (51). As a result, sodium and water reabsorption is decreased, and salt and water excretion are increased, leading to polyuria (15, 51).

AC chemotherapy may cause gastrointestinal toxicity and damage the intestinal mucosa resulting in nausea, vomiting, mucositis, diarrhea, and sometimes gastric reflux. Continued vomiting and diarrhea could lead to electrolyte imbalances (dyselectrolytemias). Chemotherapy-induced nausea results from the release of serotonin from the enterochromaffin cells, which line the GI tract (34). Serotonin then stimulates 5-HT3 receptors located in the GIT, the nucleus tractussolitarius of the medulla oblongata, and the chemoreceptor trigger zone, sending impulses to the vomiting center (34).

The mean serum potassium level was significantly increased in AC-treated BC patients. This is in agreement with a study conducted previously (21). The possible reason for the increment in the serum potassium levels among AC-treated BC patients might be the reduction of urinary potassium excretion. AC-induced tumor lysis syndrome could also have a certain effect on the elevation of serum potassium level in patients treated with AC- Therapy. AC-induced tumor lysis is characterized by hyperkalemia, hyperuricemia, hyperphosphatemia, and hypocalcemia (52, 53). Overburden of the body’s homeostatic mechanisms and devastating the capacity of the kidneys for normal excretion of potassium increased the serum potassium level (54). The rapid and massive destruction of tumor cells could lead to a subsequent release of cellular breakdown products sufficient to overwhelm the excretory mechanism and the kidneys’ normal functional capacity. The significant increment in mean potassium value may also be due to AC treatment-induced adrenal insufficiency and reduction of aldosterone section. Reduction of aldosterone secretion results in higher excretion of sodium and retention of potassium in the blood that increases potassium level in the blood (55).

Furthermore, hematological and electrolyte changes between AC- treated and untreated patients were observed in both lower and advanced stages of BC. This indicates that regardless of the stages of the disease, AC treatment affected hematological indices and serum electrolytes. However, another study showed that chemotherapy treatment in BC patients at an advanced stage showed positive outcomes (56). Study design and sample size variation between these two studies could attribute to the difference in the findings in that a prospective study design with a sample size of 47 BC patients was used in the previous study, while a cross-sectional study design on a sample size of 200 BC patients was used in our study.

## 5 Limitations of the study

In this study, financial constraints were one of the limitations in that we only analyzed three electrolytes in this study. Besides, since we have used a cross-sectional study decision, we couldn’t assess the incidence and causal inferences.

## 6 Conclusion

Total white cells, neutrophils, lymphocytes, and red blood cells are among the hematological indices reduced by AC chemotherapy treatment in patients with BC. Contrarily, eosinophils, platelets, Plateletcrit, and red cell distribution width were increased in BC patients treated with AC-chemotherapy. Besides, serum sodium was also reduced, while serum potassium was increased.

## 7 Recommendation

Routine analysis of those parameters in patients with BC is important to manage the problem. Further advanced studies incorporating other serum electrolytes and the mechanism of AC treatment-induced toxicity on hematological indices and serum electrolyte actions are required for comprehensive management. A prospective study design could be informing a more reliable result.

## Data availability statement

The original contributions presented in the study are included in the article/supplementary material. Further inquiries can be directed to the corresponding author.

## Ethics statement

The studies involving human participants were reviewed and approved by Department of medical Physiology. The patients/participants provided their written informed consent to participate in this study.

## Author contributions

Preparation of the data collection sheet, training, and close flow up of data collectors during data collection, analysis, and writing was made by FT. The research was supervised by AL in that intuitive and constructive comments were given by him. Manuscript writing and reviewing were also made by FT and AL. All authors contributed to the article and approved the submitted version.
